# An Extended, Trust-Wide Audit Assessing the Handover Process Between Mental Health Inpatient Services and Emergency Departments

**DOI:** 10.1192/bjo.2025.10616

**Published:** 2025-06-20

**Authors:** Rebecca Joy, Rachael Elliott, Seonaid Beaumont, Femi Osukoya, Sumayyah Khan

**Affiliations:** 1 Rotherham NHS Foundation Trust, Rotherham, United Kingdom; 2 Sheffield Teaching Hospitals NHS Foundation Trust, Sheffield, United Kingdom; 3 Rotherham Doncaster and South Humber NHS Foundation Trust, Scunthorpe, United Kingdom; 4 Rotherham Doncaster and South Humber NHS Foundation Trust, Rotherham, United Kingdom; 5 Rotherham Doncaster and South Humber NHS Foundation Trust, Doncaster, United Kingdom

## Abstract

**Aims:** A primary audit revealed widespread non-compliance with NICE Quality Standard (QS174) in patient handovers between the emergency department and a psychiatry inpatient unit. This second audit evaluated whether the issue was specific to one ward and/or emergency department or if it was prevalent across the trust.

**Methods:** 9 wards across 3 locations within the same trust were sampled. For each ward, online case notes from 5 patients transferred to the emergency department between April and September 2024 were reviewed, identified by selecting the most recent admissions and working backward until 5 cases were obtained. Cases were assessed for (a) handover documentation, (b) discharge summary availability, (c) required actions for the psychiatry ward, and (d) nature and implementation time of these actions. Results were analysed using Microsoft Excel.

**Results:** 45 patient case notes were reviewed. 62% (n=28) did not have a handover documented which followed the NICE definition of Situation Background Assessment Recommendation (SBAR) and 28% (n=8) had no discharge summary. 5 patients had no discharge summary and no documented handover. 56% (n=25) of patients returned with actions for the ward, and 20% (n=5) of these had actions delayed, which included medication changes (antibiotics). All but one ward identified potential issues with handovers, both between physical and mental health trusts and within the mental health trust itself.

**Conclusion:** The results of this extended audit show improvement from previous findings but still highlight significant concerns. It is possible that the number of cases reviewed were too small to detect the extent of the issue within wards. Nevertheless, the audit highlights ongoing communication issues between physical and mental health services, requiring further investigation. It also identifies the need for improvement in internal communication, as some patients with discharge summaries still experienced delayed actions.

The identification of problems across multiple sites also suggest audits of other mental health trusts would be worthwhile to establish if this is a national problem.

Quality improvement work is being undertaken to better understand and address the specific challenges faced by wards and emergency services that are affecting handovers of care across different locations within the trust. This will allow us to improve patient safety across the trust.

